# A fetus with Bosch-Boonstra-Schaaf optic atrophy syndrome characterized by bilateral ventricle widening: A case report and related literature review

**DOI:** 10.1097/MD.0000000000030558

**Published:** 2022-10-07

**Authors:** Yu Sun, Lili Guo, Jing Sha, Huimin Tao, Xuezhen Wang, Ying Liu, Jingfang Zhai, Jiebin Wu, Yongxiu Zhao

**Affiliations:** a Graduate School of Xuzhou Medical University, Jiangsu Xuzhou, China; b Department of Prenatal Diagnosis Medical Center, Xuzhou Central Hospital, Xuzhou Clinical School of Xuzhou Medical University, Xuzhou, Jiangsu, China; c Department of obstetrics, Fengxian People’s Hospital, Xuzhou, Jiangsu, China; d Graduate School of Bengbu Medical College, Bengbu, Anhui, China; e Department of laboratory, Taixing Maternity and Child Health Care Hospital, Taixing, Jiangsu, China.

**Keywords:** fetal BBSOAS, management, molecular genetics, NR2F1

## Abstract

Bosch–Boonstra–Schaaf optic atrophy syndrome (BBSOAS) is a rare neurodevelopmental disorder caused by loss-of-function variants in the Nuclear Receptor Subfamily 2 Group F Member 1 (NR2F1). Here, we report a case of fetal BBSOAS. The fetus is typically featured by bilateral ventricle widening in the late second trimester, meanwhile, a 7.94-Mb deletion fragment on 5q14.3q15 involving the whole NR2F1 gene was confirmed by copy number variation sequencing (CNV-Seq) combined with karyotyping analysis. Our aim is to provide comprehensive prenatal clinical management strategy for fetal BBSOAS.

**Patient concerns:**

A 29-year-old primipara and her husband were referred to our prenatal diagnosis center due to the widening of bilateral ventricles at 29 + 1 weeks of gestation age.

**Diagnoses:**

Ultrasound revealed the fetal widening posterior horns of bilateral ventricles at the GA of 27 + 3 weeks, 11 mm on the left and 10 mm on the right. At the following 29 + 1 weeks, ultrasound showed the posterior horn of the left lateral ventricle: 12 mm while the width of the right decreased to 9 mm, and intracranial arachnoid cyst. Furthermore, MRI confirmed that intracranial cyst might originate from an enlarged cisterna venae magnae cerebri, with mild dilation of 13.5 mm on the left ventricle. The fetal karyotyping analysis and CNV-Seq detection confirmed a 7.94-Mb deleted fragment on 5q14.3q15 (89340000_97280000) through the amniocentesis at 29 + 4 weeks of GA.

**Interventions:**

The fetus was closely monitored and underwent the following assessment by the multidisciplinary team.

**Outcomes:**

The pregnancy was terminated in the end.

**Lessons:**

It is vital to use molecular and cytogenetical detections combined with a dynamic development history to make a definite diagnosis and evaluate the genetic status for the fetuses with BBSOAS.

## 1. Introduction

BBSOAS (OMIM 615722) is an autosomal dominant disorder resulted from the muation of NR2F1, which is characterized by optic nerve atrophy (ONA), intellectual disability (ID), developmental delay (DD) and so on.^[1,2]^In clinical practice, BBSOAS is easily diagnosed after birth based on molecular genetic testing combined with specific phenotypes. However, fetal BBSOAS is relatively difficult to diagnose due to atypical phenotypes, fetal dynamic development, and difficult physical examination. In our study, fetal BBSOAS which was confirmed by genetic testing presented with bilateral ventricular widening at 27 + 3 weeks of gestation age (GA). The pregnancy was terminated in the end. Therefore, it is helpful for clinicians to use molecular genetic testing to assess the genetic status of fetus with BBSOAS and to provide the subsequent genetic counseling.

## 2. Case presentation

**Figure 1.**
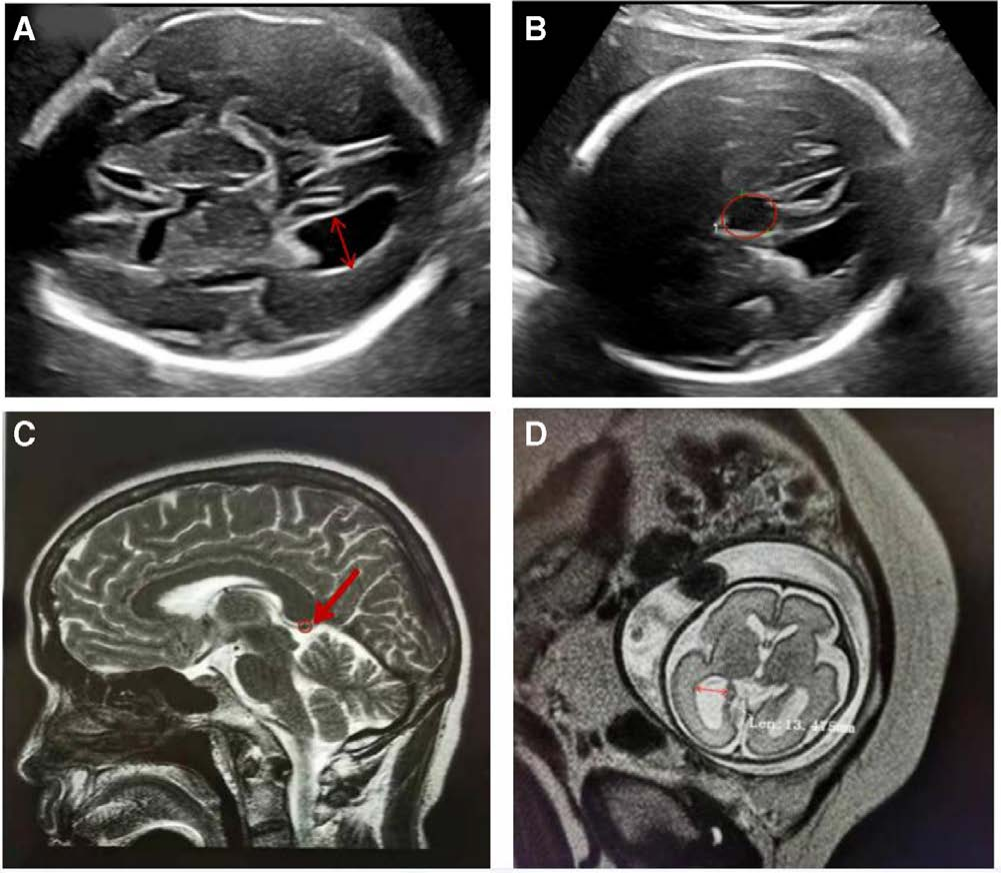
The fetal imaging results at 29 + 1 weeks of GA: (a) ultrasound showed the widening posterior horn of the left lateral ventricle (red arrow); (b) ultrasound showed intracranial arachnoid cystic structure (red circle); (c) midsagittal T2-weighted MRI image showed intracranial cyst arose from the enlarged cisterna venue magnate cerebri; (d) transverseT2-weighted MRI showed mild expansion of left ventricle.

**Figure 2.**
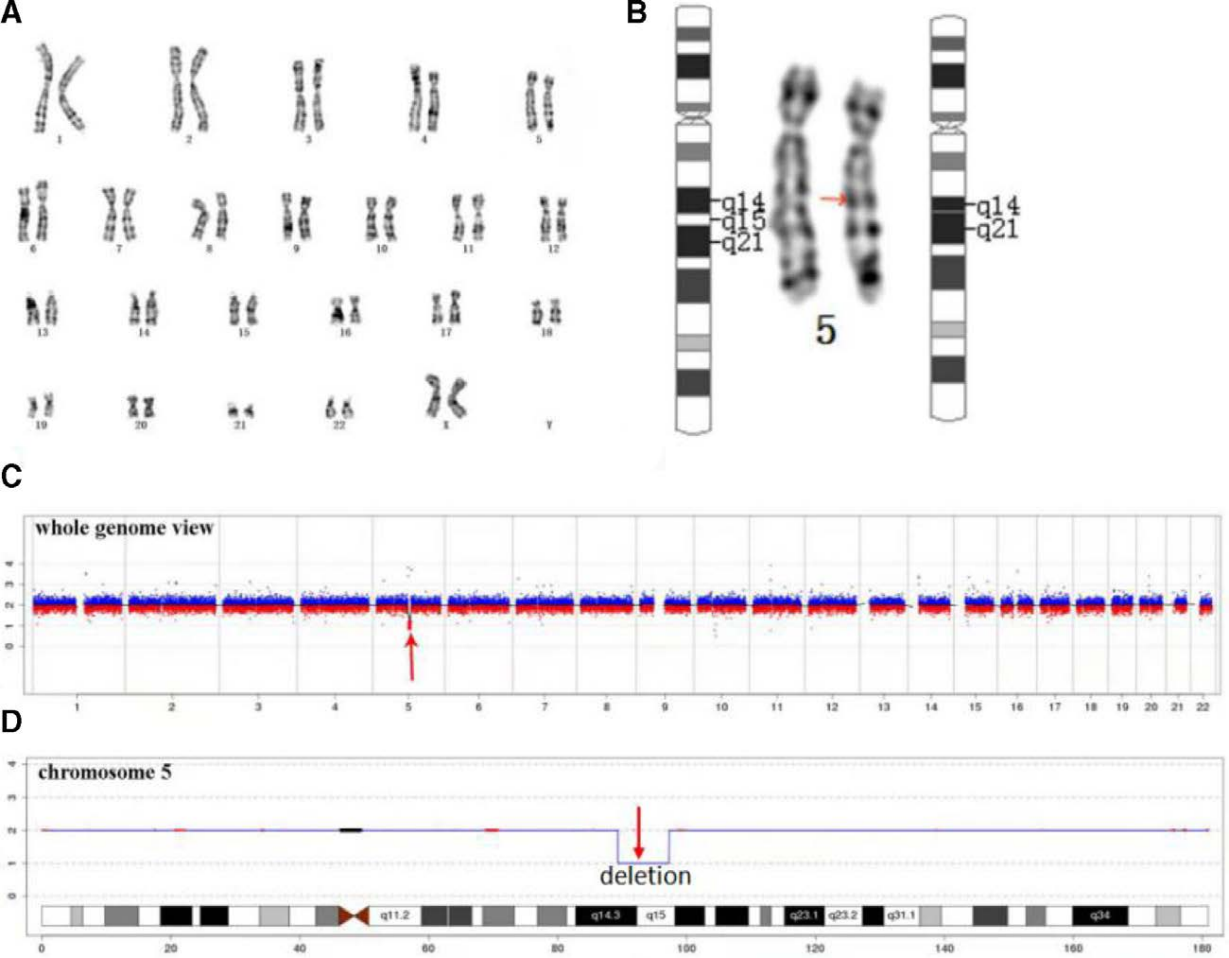
The fetal karyotype and CNV-Seq results: (a) 46, XX, del(5)(q14) at the level of 300 to 400 bands; (b) chromosome 5 and the deletion fragment of chromosome 5 (red arrow); (c) and (d) the fetal CNV-Seq result showed a 7.94-Mb deletion on 5q14.3q15 (89340000_97280000).

A 29-year-old primipara and her husband were referred to prenatal diagnosis medical center of Xuzhou Central Hospital due to the widening of bilateral ventricles at 29 + 1 weeks of GA. The couple were nonconsanguineous, healthy without any histories of drugs, infections or genetic diseases. The fetal ultrasonic screening was normal in the first trimester and the result of noninvasive prenatal screening (NIPS) was at low risk at the 18-week GA. Prenatal ultrasound from another tertiary hospital revealed the fetal widening posterior horns of bilateral ventricles at the GA of 27 + 3 weeks, 11 mm on the left and 10 mm on the right. At the following 29 + 1 weeks, ultrasound in our hospital showed the posterior horn of the left lateral ventricle: 12 mm (Fig. 1a), while the width of the right decreased to 9 mm, and intracranial arachnoid cyst was found (Fig. 1b). Furthermore, MRI confirmed that intracranial cyst might originate from an enlarged cisterna venae magnae cerebri (Fig. 1c), with mild dilation of 13.5 mm on the left ventricle (Fig. 1d). The following amniocentesis was performed at 29 + 4 weeks of GA for fetal karyotyping analysis and CNV-Seq detection after fully written consent. The fetal karyotype was 46, XX, del(5)(q14) (Fig. 2a and b)). Moreover, the fetal CNV-Seq result showed a 7.94-Mb deleted fragment on 5q14.3q15 (89340000_97280000) (Fig. 2c and d) (Human Genome Reference Assembly GRCh37/hg19). Therefore, based on molecular genetic analysis combined with ultrasound changes, the fetus was diagnosed with BBSOAS. In the end, the couple chose to terminate the pregnancy after receiving thorough genetic counseling and being well-informed of fetal possible severe adverse outcomes after labor. This process was approved by Xuzhou Central Hospital Ethics Committee. Informed consent was obtained from the patient for the publication of our case.

## 3. Discussion

BBSOAS is a rare neurodevelopmental disorder with the prevalence of less than one in one million infants.^[3–5]^ It has been confirmed to be related with the loss of function mutations of NR2F1 (OMIM 132890, NM_005654:c.286A > G:p.Lys96Glu).^[1,6]^ NR2F1, as a highly orphan nuclear receptor and transcriptional regulator, is composed of ligand inducible transcription factors and plays a vital role in neurogenesis and neural crest cell differentiation.^[7]^ This NR2F1gene in our case was assessed as haploinsufficiency (gnomad pLI score > 0.9 and upper limit of O/E confidence interval < 0.35, decipher %HI < 10) by the haploidy under-dose prediction tool in the GNOMAD and Decipher websites. Furthermore, postnatal hypomyelination and astrogliosis are confirmed to be related with an imbalance development between oligodendrocytes and astrocytesin in NR2F1-deficient optic nerves of mice.^[8]^ The NR2F1 haploinsufficiency leads to related protein changes in the zinc-finger DNA-binding domain, thus impedes its transcriptional activity,^[3,4]^which may contribute to better explain OA, DD, ID, and other related clinical phenotypes.^[6]^ The majority of BBSOAS have optic dysplasia, often accompanied by brain structural abnormalities related to SOX2 gene (OMIM 184429).^[9]^ In the present study, the fetus presented with bilateral mild ventriculomegaly (VM) and intracranial cyst by imaging examinations in the late second and early third trimester, which may originate from the above mutations of NR2F1. However, it still remains to be verified that NR2F1 is associated with hydrocephalus using a lot of clinical data or related animal research results. Fortunately, the couple accepted amniocentesis for genetic causes of the fetus. The traditional karyotyping combined CNV-Seq confirmed a fetal novel 7.94-Mb deletion on 5q14.3q15 (89340000_97280000) × 1, comprising heterozygous NR2F1 mutation. In addition, other disease-associated genes in this region are autosomal recessive genes including adhesion G protein-coupled receptor V1 (ADGRV1), arylsulfatase K (ARSK), calpastatin (CAST), kiaa0825 gene (KIAA0825), proprotein convertase, subtilisin/kexin-type 1 (PCSK1), tetratricopeptide repeat domain-containing protein 37 (TTC37). Hence, the precise localization of CNV-Seq in chromosomal fragment may contribute to genetic cause of fetal intracranial phenotypes and diagnose fetal BBSOA. So far, a total of 44 patients with BBSOAS including our case are counted with heterozygous NR2F1 variants, 43 individuals in the related literatures (Table 1),^[3–6,10–17]^ of which 35 patients exist point mutations or inframe deletions or insertions in the DBD, 9 individuals present with the deletion fragments on the chromosme 5q14.3q15 including NR2F1 gene and the range of deleted sizes from 0.2 to 7.94 Mb. In terms of age, the range is relatively wide from the 32-week fetus to the 43-year-old adult. However, the deletion fragment of our fetus is larger than those of reported cases. Furthermore, the table demonstrates 39 of 43 cases derived from de novo mutations, which means that the majority of BBSOAS cases occur accidentally. Hence, molecular diagnostic techniques are critical to early identify the cases with BBSOAS.

**Table 1:** The spectrum of clinical phenotypes, NR2F1 variants and the deletion fragments on the chromosme 5q including NR2F1 with BBSOAS.

Cases	Literature	publication date	Mutations	Sex	Age	Inheritance	DD/ID	ONA	Hypotonia	Oromotor dysfunction	Thin corpus callosum	Repetitive behavior	ASD	Seizure	Febrile seizure	ADHD	Hearing defect	Spasticity
1	4	2014	c.339C > A; p.Ser113Arg	F	2y4m	de novo	+	+	N/A	N/A	N/A	N/A	N/A	N/A	N/A	N/A	N/A	+
2	4	2014	c.344G > C; p.Arg115Pro	M	12y	de novo	+	+	+	N/A	N/A	–	N/A	N/A	N/A	N/A	N/A	N/A
3	4	2014	c.755T > C; p.Leu252Pro	F	18y	de novo	+	+	+	N/A	N/A	N/A	N/A	N/A	N/A	N/A	N/A	N/A
4	4	2014	c.335G > A; p.Arg112Lys	F	35y	de novo	+	–	N/A	N/A	N/A	N/A	+	N/A	N/A	N/A	N/A	–
5	3	2016	c.2T > C; p.M1?	F	3y	de novo	+	CVI	+	–	N/A	–	–	–	–	–	–	–
6	3	2016	c.403C > A; p.R135S	F	4y	de novo	+	CVI	+	+	+	–	–	IS	–	–	–	–
7	3	2016	c.425G > T; p.R142L	F	4y	de novo	+	+	+	+	+	–	–	+	–	–	–	–
8	3	2016	c.2T > G; p.M1?	F	4y	de novo	+	+	–	+	+	–	–	–	–	–	–	–
9	3	2016	c.328_330del; p.Phe110del	F	6y	de novo	+	+	+	–	+	–	–	IS	–	–	–	–
10	3	2016	c.382T > C; p.C128R	M	6y	de novo	+	+	+	+	+	+	+	+	–	–	+	–
11	3	2016	c.2T > G; p.M1?	M	7y	de novo	+	+	+	+	–	+	+	–	–	–	+	+
12	3	2016	c.463G > A; p.A155T	M	10y	de novo	–	–	+	–	–	–	–	–	–	–	–	–
13	3	2016	c.436T > C; p.C146R	M	11y	de novo	+	+	+	+	+	+	–	–	–	–	–	–
14	3	2016	c.2T > C; p.M1?	F	12y	de novo	+	+	+	+	+	+	–	+	–	+	+	–
15	3	2016	c.413G > A; p.C138Y	M	15y	de novo	+	+	–	–	–	+	+	–	+	–	–	–
16	3	2016	c.1103G > A; p.G368D	M	21y	de novo	+	–	–	–	–	+	+	+	–	–	–	–
17	3	2016	c.291delC; p.His79Hisfs *22	M	21y	de novo	+	+	+	+	N/A	+	+	–	–	+	–	–
18	3	2016	c.103_113delinCGCCGCCGC; p.Gly35Argfs *361	M	30y	de novo	+	+	+	+	–	+	+	+	–	+	+	–
19	3	2016	c.2_4delinTGG; p.M1?	M	43y	de novo	+	+	+	+	–	–	–	+	–	–	–	–
20	10	2017	c.1115T > C; L372P	F	15m	de novo	+	N/A	+	+	N/A	+	N/A	N/A	N/A	N/A	–	N/A
21	10	2017	c.257G > T; cys86phe	M	14y	de novo	+	+	+	+	N/A	N/A	N/A	+	N/A	+	N/A	N/A
22	11	2017	c.403C > T; p.R135C	F	23y	de novo	+	+	–	–	N/A	N/A	+	N/A	–	N/A	N/A	+
23	12	2018	c.286A > G: p.Lys96Glu	F	17y	de novo	+	+	+	N/A	+	N/A	N/A	+	–	N/A	N/A	+
24	13	2019	c.513C > G; p.Tyr171Ter	M	7y	de novo	+	+	N/A	N/A	N/A	N/A	–	N/A	N/A	+	N/A	+
25	14	2020	c.425G > A;p.Arg142His	F	1y4m	de novo	+	+	+	–	+	–	–	–	–	–	–	–
26	14	2020	c.2T > C;p.?	F	3y5m	de novo	+	–	–	N/A	+	N/A	+	–	–	–	+	+
27	14	2020	c.729730 delinsCT;p.Gln244*	M	3y7m	de novo	+	+	+	N/A	+	N/A	–	–	–	N/A	–	–
28	14	2020	c.115G > T; p.Glu39*	F	5y10m	de novo	+	+	+	N/A	+	–	+	N/A	+	+	–	–
29	14	2020	c.292T > C; p.Tyr98His	F	6y7m	de novo	+	+	–	N/A	+	N/A	+	–	–	–	–	+
30	14	2020	c.967_968delAA; p.Lys323Serfs*73	M	12y2m	de novo	+	+	N/A	+	–	N/A	N/A	N/A	N/A	N/A	N/A	N/A
31	15	2020	c.1080del; p.Asn362fs*33	M	9y	de novo	+	+	+	–	+	N/A	+	–	+	N/A	+	+
32	16	2020	c.82C > T; p.Gln28*	M	23y	de novo	–	+	+	+	N/A	–	+	N/A	N/A	+	+	+
33	17	2020	c.253 G > T; p.E85X	M	32y	de novo	+	+	N/A	N/A	N/A	N/A	N/A	+	N/A	N/A	N/A	N/A
34	5	2020	c.313G > A; p.Gly105Ser	N/A	16y	de novo	+	+	N/A	N/A	N/A	+	N/A	+	N/A	N/A	N/A	+
35	5	2020	c.313G > A; p.Gly105Ser	N/A	16y	de novo	+	+	N/A	N/A	N/A	+	N/A	+	N/A	N/A	N/A	+
36	6	2013	Deletion (0.582 Mb)	M	8y3m	de novo	+	+	+	+	N/A	N/A	N/A	–	N/A	+	+	+
37	4	2014	Deletion (2.85 Mb)	F	4y	de novo	+	CVI	+	N/A	N/A	N/A	N/A	N/A	N/A	N/A	N/A	+
38	4	2014	Deletion (0.83 Mb)	F	24y	N/A	+	CVI	N/A	N/A	N/A	N/A	N/A	N/A	N/A	N/A	N/A	N/A
39	3	2016	Deletion (0.9 Mb)	M	2y	paternal	+	+	+	+	+	–	–	–	–	–	–	–
40	3	2016	Deletion (5.0 Mb)	F	6y	N/A	+	–	+	+	–	–	–	–	–	–	–	–
41	3	2016	Deletion (0.2 Mb)	F	8y	de novo	+	+	+	–	N/A	–	–	–	–	+	–	–
42	3	2016	Deletion (0.9 Mb)	M	35y	N/A	+	+	–	–	N/A	–	–	–	–	+	–	–
43	3	2016	Deletion (1.2 Mb)	M	37y	N/A	+	+	–	–	N/A	–	+	–	–	–	–	–
44	our case	2021	Deletion (7.94 Mb)	F	32w	de novo	N/A	N/A	N/A	N/A	–	N/A	N/A	N/A	N/A	N/A	N/A	N/A

N/A = not available.

In addition to disturbance of cerebrospinal fluid circulation and fetal brain infections, VM may be associated with cortical dysplasia. The subtle cortical malformations were revealed in the patients with BBSOAS and the cortical abnormality was confirmed in BBSOAS mouse model to be associated with an NR2F1 gene.^[14]^ It is mentioned in particular that the molecular genetic testing is essential to identically further clarify the diagnosis of fetuses with suspected septo-optic dysplasia and other abnormal structures close to midline in the brain, which might be a series of milestones of early fetal brain development manifestations related to BBSOAS. The brain MRI results reported in the literature showed that corpus callosum thinning was observed in 8 of 15 BBSOAS patients, all presenting with DD, ID, and optic nerve atrophy (ONA), while DD and ID were observed in 6 of 7 normal corpus callosum patients with BBSOAS, and ONA was observed in 4 of them.^[3]^ Furthermore, Armentano M revealed the important role and mechanism of NR2F1 in the abnormal development of corpus callosum during axon growth in vivo mouse.^[18]^ However, our fetus was lack of corpus callosum thinning which might be related to the small GA.

Based on clinical symptoms, physical examination, imaging evaluation, molecular genetics as well as cytogenetics, fetal BBSOAS can be effectively diagnosed. However, most optic nerve abnormalities and some intracranial abnormalities during fetal period could not be detected due to fetal immature development. And subtle phenotypic changes cannot be entirely detected by ultrasound or MRI. In addition, some fetal symptoms of BBSOAS such as DD and ID can not be assessed accurately until the neonatal period or even adulthood. All of the above factors increase the difficulty of fetal BBSOAS diagnosis during the pregnancy. In actual clinical practice, even if fetal intracranial structural abnormalities are found by ultrasound scanning in the second or third trimester of pregnancy, some pregnant women would rather choose dynamic ultrasound examination to monitor the structural changes than accept invasive prenatal diagnosis to avoid the risk of abortion.^[19]^ Therefore, fetal BBSOAS is always easily overlooked. The couple in our report accepted and underwent amniocentesis And the fetal BBSOAS was further diagnosed definitely. Hence, the birth of the defective fetus was prevented. Therefore, once ultrasound detects fetal VM or other intracranial abnormal structures, it is important to obtain a detailed case history, such as viral diseases, trauma, family history, fetal thrombocytopenia, etc. At the same time, it is more necessary to provide further CNV-Seq and/or whole exome sequencing (WES) gene detections for the fetuses with abnormal structures.^[20]^ Here, it is worth mentioning for clinicians that invasive prenatal diagnosis for molecular genetics should be recommended for those families with a history of BBSOAS or fetal intracranial structural changes, or 5q deletion fragment including NR2F1 indicated by NIPS. Therefore the diagnosis for fetal BBSOAS relies on the dynamical information in detail from ultrasound and magnetic resonance, meanwhile, appropriate genetic testing is also vital to help the clinicians determine the diagnosis and provide the reasonable counseling.

In conclusion, it is necessary to use molecular and cytogenetical detections combined with a dynamic development history to make a definite diagnosis and evaluate the genetic status for the fetuses with BBSOAS.

## Acknowledgments

We would like to thank for the couple’s participation and the staff’s cooperation of the department of Prenatal Diagnosis Medical Center and Ultrasound of Xuzhou Central Hospital.

## Author contributions

Jingfang Zhai, Yu Sun and Lili Guo performed the conception, acquisition of data, and were major contributors in writing the manuscript. All authors analyzed and interpreted the patient data regarding the ultrasound screening and CNV-Seq, read and approved the final manuscript.
